# The Transcription Factor Mrr1p Controls Expression of the *MDR1* Efflux Pump and Mediates Multidrug Resistance in *Candida albicans*

**DOI:** 10.1371/journal.ppat.0030164

**Published:** 2007-11-02

**Authors:** Joachim Morschhäuser, Katherine S Barker, Teresa T Liu, Julia Blaß-Warmuth, Ramin Homayouni, P. David Rogers

**Affiliations:** 1 Institut für Molekulare Infektionsbiologie, Universität Würzburg, Würzburg, Germany; 2 Department of Clinical Pharmacy, University of Tennessee Health Science Center, Memphis, Tennessee, United States of America; 3 Children's Foundation Research Center at Le Bonheur Children's Medical Center, Memphis, Tennessee, United States of America; 4 Bioinformatics Program, Department of Biology, University of Memphis, Memphis, Tennessee, United States of America; 5 Department of Pharmaceutical Sciences, University of Tennessee Health Science Center, Memphis, Tennessee, United States of America; 6 Department of Pediatrics, University of Tennessee Health Science Center, Memphis, Tennessee, United States of America; 7 Department of Molecular Sciences, University of Tennessee Health Science Center, Memphis, Tennessee, United States of America; University of California San Francisco, United States of America

## Abstract

Constitutive overexpression of the *MDR1* (multidrug resistance) gene, which encodes a multidrug efflux pump of the major facilitator superfamily, is a frequent cause of resistance to fluconazole and other toxic compounds in clinical Candida albicans strains, but the mechanism of *MDR1* upregulation has not been resolved. By genome-wide gene expression analysis we have identified a zinc cluster transcription factor, designated as *MRR1* (multidrug resistance regulator), that was coordinately upregulated with *MDR1* in drug-resistant, clinical C. albicans isolates. Inactivation of *MRR1* in two such drug-resistant isolates abolished both *MDR1* expression and multidrug resistance. Sequence analysis of the *MRR1* alleles of two matched drug-sensitive and drug-resistant C. albicans isolate pairs showed that the resistant isolates had become homozygous for *MRR1* alleles that contained single nucleotide substitutions, resulting in a P683S exchange in one isolate and a G997V substitution in the other isolate. Introduction of these mutated alleles into a drug-susceptible C. albicans strain resulted in constitutive *MDR1* overexpression and multidrug resistance. By comparing the transcriptional profiles of drug-resistant C. albicans isolates and *mrr1*Δ mutants derived from them and of C. albicans strains carrying wild-type and mutated *MRR1* alleles, we defined the target genes that are controlled by Mrr1p. Many of the Mrr1p target genes encode oxidoreductases, whose upregulation in fluconazole-resistant isolates may help to prevent cell damage resulting from the generation of toxic molecules in the presence of fluconazole and thereby contribute to drug resistance. The identification of *MRR1* as the central regulator of the *MDR1* efflux pump and the elucidation of the mutations that have occurred in fluconazole-resistant, clinical C. albicans isolates and result in constitutive activity of this trancription factor provide detailed insights into the molecular basis of multidrug resistance in this important human fungal pathogen.

## Author Summary

The *Candida albicans MDR1* (multidrug resistance) gene encodes a multidrug efflux pump of the major facilitator superfamily that is constitutively overexpressed in many fluconazole-resistant strains. Although *MDR1* overexpression is a major cause of resistance to this widely used antifungal agent and other metabolic inhibitors, so far the molecular basis of *MDR1* upregulation in resistant strains has remained elusive. By comparing the transcription profiles of *MDR1* overexpressing, clinical C. albicans isolates and matched, drug-susceptible isolates from the same patients, we identified a transcription factor, termed multidrug resistance regulator 1 (*MRR1*), which was upregulated in all resistant isolates and turned out to be a central regulator of *MDR1* expression. Resistant isolates contained point mutations in *MRR1*, which rendered the transcription factor constitutively active. Introduction of these mutated alleles into a susceptible strain caused *MDR1* overexpression und multidrug resistance. Inactivation of *MRR1* in clinical isolates abolished *MDR1* expression and affected fluconazole resistance even more strongly than deletion of the *MDR1* efflux pump itself, indicating that additional Mrr1p target genes, which were identified by genome-wide gene expression analysis, contribute to fluconazole resistance. These findings provide detailed insights into the molecular basis of multidrug resistance in one of the most important human fungal pathogens.

## Introduction

The yeast Candida albicans is usually a harmless commensal in many healthy people where it resides on mucosal surfaces of the gastrointestinal and urogenital tract, but it can also cause superficial as well as life-threatening systemic infections, especially in immunocompromised patients [1]. Infections by C. albicans are commonly treated with the antimycotic agent fluconazole that inhibits the biosynthesis of ergosterol, the major sterol in the fungal cell membrane. However, C. albicans can develop resistance to fluconazole, especially during long-term treatment of oropharyngeal candidiasis, which frequently affects HIV-infected persons and AIDS patients [2]. Molecular fingerprinting of serial C. albicans isolates from recurrent episodes of oropharyngeal candidiasis has shown that fluconazole resistance usually develops in previously susceptible strains, and such serial isolates from the same patient, so-called matched isolates, have proved an excellent tool to study the molecular basis of drug resistance [3–10]. Fluconazole resistance can be caused by different mechanisms, including alterations in the sterol biosynthetic pathway, increased expression of the *ERG11* gene that encodes the target enzyme of fluconazole, sterol 14α-demethylase (Erg11p), mutations in the *ERG11* gene that result in reduced affinity of Erg11p to fluconazole, and overexpression of genes encoding membrane transport proteins, which transport fluconazole out of the cell. In clinical C. albicans strains, several of these mechanisms are often combined to result in a stepwise development of clinically relevant fluconazole resistance (for a review, see [11]).

A major mechanism of drug resistance in C. albicans is the constitutive upregulation of genes encoding efflux pumps that actively transport fluconazole and many other, structurally unrelated toxic compounds out of the cell. Two types of efflux pumps have been identified in C. albicans [12–15]. The *CDR1* and *CDR2* genes encode ATP-binding cassette (ABC) transporters, whereas *MDR1* encodes a multidrug efflux pump of the major facilitator superfamily. In drug-susceptible C. albicans strains, *MDR1* is expressed at low or non-detectable levels in standard laboratory media, but its expression can be induced when the cells are grown in the presence of certain toxic compounds, like benomyl, hydrogen peroxide, or diamide [16–20]. In contrast, many fluconazole-resistant, clinical C. albicans isolates constitutively overexpress *MDR1* [3,4,7–10]. Inactivation of *MDR1* in such *MDR1* overexpressing C. albicans isolates increased their susceptibility to fluconazole, confirming that *MDR1* overexpression contributed to fluconazole resistance [21]. The increased resistance of such isolates to other metabolic inhibitors, like cerulenin, brefeldin A, and diamide was completely abolished after *MDR1* deletion, indicating that the resistance of the strains to these toxic compounds was mainly or exclusively mediated by Mdr1p [22,23]. Comparison of the *MDR1* promoter sequences in matched pairs of fluconazole-susceptible and *MDR1* overexpressing, fluconazole-resistant C. albicans isolates from the same patient demonstrated that the constitutive *MDR1* upregulation in the resistant isolates was not caused by promoter mutations but by alterations in *trans*-regulatory factor(s) [24]. Several groups have identified sequences in the *MDR1* promoter region that mediate its upregulation in drug-sensitive strains in response to inducing chemicals and its constitutive activation in drug-resistant strains [19,20,25,26]. However, in contrast to the ABC transporters *CDR1* and *CDR2*, whose expression has recently been shown to be controlled by the transcription factor Tac1p, which is mutated in *CDR1*/*CDR2* overexpressing C. albicans strains [27,28], the regulatory factors controlling *MDR1* expression and the mutations that are responsible for its constitutive overexpression in drug-resistant clinical isolates have not yet been identified.

In this study, we compared the alterations in gene expression occurring in three different *MDR1* overexpressing, clinical C. albicans isolates on a genome-wide scale to identify genes that are commonly upregulated with *MDR1*. This approach led to the identification of a central regulator of *MDR1* expression and to the elucidation of the molecular basis of *MDR1* overexpression and multidrug resistance in clinical C. albicans isolates.

## Results

### Expression of the Transcription Factor *MRR1* Is Coordinately Upregulated with *MDR1* in Drug-Resistant, Clinical C. albicans Isolates

**Figure 1:**
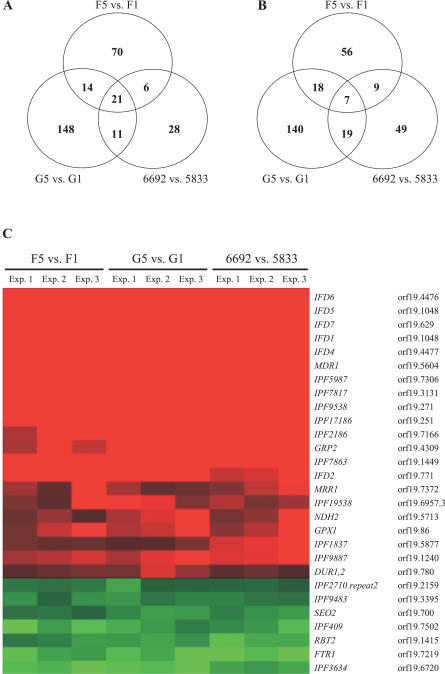
Identification of Genes that Are Differentially Expressed in *MDR1* Overexpressing Clinical C. albicans Isolates (A, B) Venn Diagrams showing the number of genes that are upregulated (A) or downregulated (B) in the *MDR1* overexpressing, drug-resistant isolates F5, G5, and 6692 as compared with their matched drug-susceptible isolates F1, G1, and 5833, respectively. (C) Heat map showing genes that are upregulated (red) or downregulated (green) in all three *MDR1* overexpressing isolates. Results are from three independent experiments (Exp. 1 to Exp. 3) for each comparison. The genes are ordered according to their relative degree of up- and downregulation, as indicated by the brightness of the red and green fields (see Table S1 for details about the expression data). Gene names were taken from CandidaDB (http://genolist.pasteur.fr/CandidaDB), except for *MDR1* and *MRR1*, and their orf19 names are given.

To identify genes that are coordinately upregulated with *MDR1*, we compared the transcriptional profiles of three matched pairs of fluconazole-susceptible and fluconazole-resistant clinical C. albicans isolates. F1 and G1 are the first, drug-susceptible isolates of two well-characterized series of clinical C. albicans isolates and do not detectably express *MDR1*, while F5 and G5 are the last isolates in each series that overexpress *MDR1* and have become multidrug-resistant [8,21,23,24]. An additional isolate pair from another patient, isolates 5833 (no *MDR1* expression) and 6692 (*MDR1* overexpression), was obtained from Martine Raymond and has been described recently [29]. As can be seen in Figure 1, a common set of 21 genes was consistently upregulated in all three *MDR1* overexpressing isolates, including all the genes encoding proteins that had previously been identified as upregulated in isolates F5 and G5 by proteome analysis [23]. In addition, seven genes were downregulated in all resistant isolates. The complete data set for all differentially expressed genes in the pairwise comparisons can be found in Table S1. Interestingly, one gene, orf19.7372 (IPF1266), which was moderately upregulated in the *MDR1* overexpressing strains, encodes a predicted zinc cluster transcription factor that has been given the preliminary name *ZCF36* in the *Candida* Genome Database (http://www.candidagenome.org/). As transcription factors often regulate their own expression in addition to that of their target genes, we hypothesized that this transcription factor might be involved in *MDR1* expression and thereby control multidrug resistance. The results shown below demonstrate that this was indeed the case and we have therefore named orf19.7372 as *MRR1*, for multidrug resistance regulator.

### Inactivation of *MRR1* Abolishes Multidrug Resistance of *MDR1* Overexpressing C. albicans Strains

**Figure 2:**
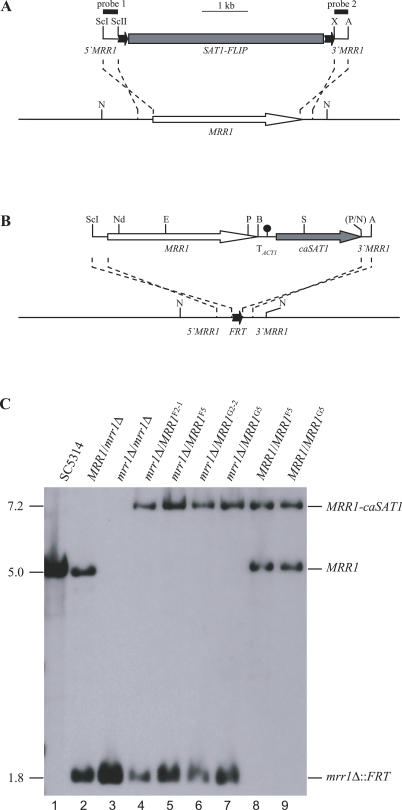
Construction of *mrr1*Δ Mutants and Complemented Strains (A) Structure of the deletion cassette from plasmid pZCF36M2 (top), which was used to delete the *MRR1* ORF in strains SC5314, F5, G5, and CAG48B, and genomic structure of the *MRR1* locus in the parental strains (bottom). The *MRR1* coding region is represented by the white arrow and the upstream and downstream regions (5′*MRR1* and 3′*MRR1*) by the solid lines. The *SAT1* flipper cassette (*SAT1-FLIP*), in which the *caFLP* gene is expressed from the inducible *SAP2* promoter [30], is represented by the grey rectangle bordered by *FRT* sites (black arrows). The 34 bp *FRT* sites are not drawn to scale. The probes used for Southern hybridization analysis of the mutants are indicated by the black bars. (B) Structure of the DNA fragments from plasmids pZCF36K2, pZCF36K3, pZCF36K4, and pZCF36K5 (top), which were used for integration the *MRR1* ^F2–1^, *MRR1* ^F5^, *MRR1* ^G2–2^, and *MRR1* ^G5^ alleles, respectively, into the disrupted *mrr1* locus of homozygous and heterozygous *mrr1*Δ mutants (bottom) using the *caSAT1* selection marker (grey arrow). T*_ACT1_*, transcription termination sequence of the *ACT1* gene. Only relevant restriction sites are given in (A) and (B): A, *Apa*I; B, *Bgl*II; E, *Eco*RI; N, *Nsi*I; Nd, *Nde*I; P, *Pst*I; S, *Sal*I; ScI, *Sac*I; ScII, *Sac*II; X, *Xho*I. The *Pst*I and *Nsi*I sites shown in parenthesis were destroyed by the cloning procedure. (C) Southern hybridization of *Nsi*I-digested genomic DNA of *mrr1*Δ mutants derived from strain SC5314 and of strains with reinserted *MRR1* alleles with the *MRR1*-specific probe 1. The sizes of the hybridizing fragments (in kb) are given on the left side of the blot and their identities are indicated on the right. The genotype of the strains is given above the respective lanes. Only one of the two independently constructed series of strains is shown. Inactivation of *MRR1* in the clinical isolates F5 and G5 and the reporter strain CAG48B and reinsertion of different *MRR1* alleles in *mrr1* mutants of the reporter strain occurred in an analogous fashion.

**Figure 3:**
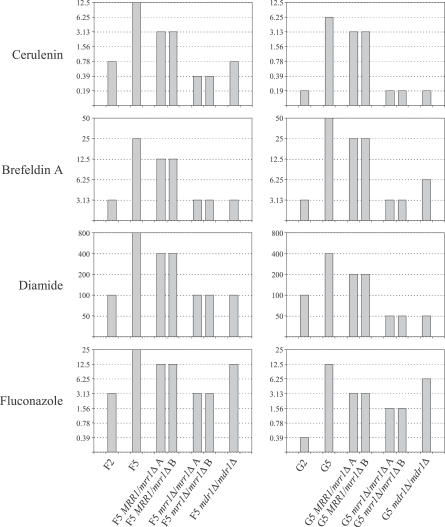
*MRR1* Mediates Drug Resistance of *MDR1* Overexpressing Clinical C. albicans Isolates Minimal inhibitory concentration (MIC) of the indicated metabolic inhibitors (in μg ml^−1^) for the drug-sensitive clinical isolates F2 and G2, the matched drug-resistant isolates F5 and G5, and two independently constructed heterozygous and homozygous *mrr1*Δ mutants as well as *mdr1*Δ mutants of the drug-resistant isolates F5 and G5. The susceptibilities of the isolates F2, F5, and its mutant derivatives are shown in the left panels, and those of isolates G2, G5, and its mutant derivatives are shown in the right panels.

To investigate if *MRR1* affects drug resistance in C. albicans, we deleted the gene in the C. albicans model strain SC5314 as well as in the drug-resistant clinical isolates F5 and G5 using the *SAT1*-flipping strategy ([30], Figure 2A and 2C, lanes 1–3, and unpublished data). From each parental strain, two independent homozygous *mrr1*Δ mutants were constructed (see Table S2) and tested for their susceptibilities to various metabolic inhibitors to which *MDR1* overexpression confers resistance [22,23,31]. Inactivation of *MRR1* in the drug-susceptible strain SC5314, which does not detectably express *MDR1* under standard growth conditions, did not affect its susceptibility to the tested compounds, except for diamide, which is known to induce *MDR1* expression (see also below). In contrast, the *mrr1*Δ mutants of the *MDR1* overexpressing isolates F5 and G5 completely lost their resistance to cerulenin, brefeldin A, and diamide and became as susceptible to these inhibitors as *mdr1*Δ mutants derived from these strains or the matched, drug-susceptible isolates F2 and G2, which were the last isolates in each series that did not detectably express *MDR1* [8] (Figure 3). The heterozygous *mrr1* mutants exhibited intermediate resistance. Resistance of isolates F5 and G5 to these compounds was mediated mostly or exclusively by *MDR1* overexpression, since resistance was lost after deletion of *MDR1* [22,23]. In contrast, *MDR1* overexpression contributes only partially to fluconazole resistance of these isolates, which is caused by a combination of different mechanisms [8,21]. Interestingly, deletion of *MRR1* in isolates F5 and G5 had an even stronger effect than inactivation of *MDR1*, suggesting that *MRR1* is required for various mechanisms of fluconazole resistance. The increased fluconazole resistance of isolate F5 as compared with isolate F2 was completely lost in the *mrr1*Δ mutants derived from this strain, while the *mrr1*Δ mutants of isolate G5, which also contains a mutation in the *ERG11* gene that results in reduced affinity of fluconazole for its target enzyme, were still more resistant than the matched isolate G2, which does not contain this mutation [8].

### *MRR1* Mediates Constitutive *MDR1* Overexpression as well as Inducible *MDR1* Expression

**Figure 4:**
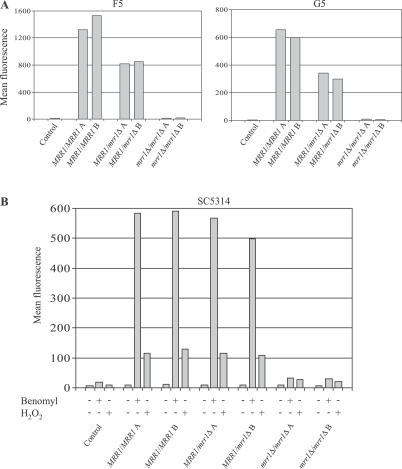
*MRR1* Is Required for Constitutive and Induced *MDR1* Expression (A) Constitutive *MDR1* promoter activity in the drug-resistant clinical isolates F5 and G5 and in heterozygous and homozygous *mrr1*Δ mutants. The mean fluorescence of two independently constructed derivatives of each parental strain carrying a P*_MDR1_-GFP* reporter fusion and grown to log phase in YPD medium was determined by flow cytometry. The parental strains F5 and G5, which do not contain the *GFP* gene, were included as negative controls. (B) Benomyl- and H_2_O_2_-induced *MDR1* promoter activity in strain SC5314 and its *mrr1*Δ derivatives. Two independently constructed reporter strains of each parental strain carrying a P*_MDR1_-GFP* reporter fusion were grown in the absence (-) or presence (+) of benomyl or H_2_O_2_ as detailed in the experimental procedures and the mean fluorescence of the cells was determined by flow cytometry. The parental strain SC5314, which does not contain *GFP*, was included to control for background fluorescence.

The results presented above suggested that *MRR1* affects drug resistance by controlling expression of the *MDR1* efflux pump. Therefore, we compared *MDR1* promoter activity in the *mrr1*Δ mutants and their wild-type parental strains. For this purpose, a P*_MDR1_-GFP* (green fluorescent protein) reporter fusion from plasmid pMPG2S (see Materials and Methods) was integrated into the genome of these strains, and *GFP* expression was quantified by flow cytometry. As can be seen in Figure 4A, the strong *MDR1* promoter activity in isolates F5 and G5 was completely abolished after deletion of *MRR1*, demonstrating that *MRR1* is required for the constitutive activation of the *MDR1* promoter in these clinical isolates. The heterozygous *MRR1*/*mrr1*Δ mutants exhibited reduced *MDR1* promoter activity, showing that both *MRR1* alleles contributed to *MDR1* overexpression in the drug-resistant isolates. The loss of *MDR1* expression in the *mrr1*Δ mutants was also independently confirmed by quantitative real-time reverse transcription (RT)-PCR (see Figure S1A).

As mentioned above, strain SC5314 does not detectably express *MDR1*, and no significant *MDR1* promoter activity was seen in the corresponding reporter strains (Figure 4B). However, *MDR1* expression can be induced in drug-susceptible strains when the cells are exposed to certain chemicals [16–19]. We used two such compounds, benomyl and H_2_O_2_, to investigate whether *MRR1* is also required for inducible *MDR1* expression. These chemicals were chosen because it has recently been reported that different regions in the *MDR1* promoter mediate its upregulation by benomyl and H_2_O_2_ [20]. While the deletion of one *MRR1* allele had no effect on *MDR1* promoter activity under these conditions, activation of the *MDR1* promoter by either of the two compounds was almost completely abolished in the homozygous *mrr1*Δ mutants (Figure 4B). Therefore, *MRR1* is required for both the constitutive *MDR1* overexpression in drug-resistant, clinical C. albicans isolates and the inducible *MDR1* expression in a drug-susceptible strain.

### The Drug-Resistant, Clinical C. albicans Isolates F5 and G5 Contain Mutated *MRR1* Alleles

Since *MRR1* was upregulated in the *MDR1* overexpressing clinical C. albicans isolates, we investigated whether artificial overexpression of *MRR1* would result in activation of the *MDR1* promoter. For this purpose, we placed the *MRR1* coding region from strain SC5314 under the control of the strong *ADH1* promoter in plasmid pZCF36E1 (see Materials and Methods). The P*_ADH1_-MRR1* fusion was integrated into strain CAG48B, a derivative of the fluconazole-sensitive laboratory strain CAI4, which expresses the *GFP* reporter gene from the endogenous *MDR1* promoter (see Table S2). The resulting strain did not detectably express the *GFP* gene, similar to a control strain that carried an otherwise identical construct without the *MRR1* gene (unpublished data), suggesting that overexpression of *MRR1* was not sufficient to activate the *MDR1* promoter and that the drug-resistant isolates F5 and G5 might carry gain-of-function mutations in *MRR1*. Therefore, we cloned and sequenced the *MRR1* alleles of isolates F2, F5, G2, and G5 (see Table S3). Isolate F2 contained two polymorphic *MRR1* alleles. The coding region of allele 1 (*MRR1* ^F2–1^) was identical to the *MRR1* sequence of strain SC5314, while allele 2 (*MRR1* ^F2–2^) differed from it at 26 positions, with three of the polymorphisms resulting in amino acid exchanges. Only one allele (*MRR1* ^F5^) was obtained from the matched resistant isolate F5 and this allele corresponded to allele 1 of isolate F2 except for a single C-T mutation at position 2047, which resulted in a proline-serine substitution at position 683 of Mrr1p. Direct sequencing of the PCR products confirmed the loss of heterozygosity in isolate F5 and the absence of the mutation in the *MRR1* alleles of isolate F2. A similar situation was found for the isolate pair G2/G5. Isolate G2 contained two polymorphic *MRR1* alleles (*MRR1* ^G2–1^ and *MRR1* ^G2–2^), which differed from one another at 28 positions, with two of the polymorphisms resulting in amino acid differences. G5 contained only one *MRR1* allele (*MRR1* ^G5^) that was identical with allele 2 of isolate G2 except for a single G-T mutation at position 2990, which resulted in a glycine–valine substitution at position 997 of Mrr1p. Therefore, both resistant isolates F5 and G5 had become homozygous for a mutated *MRR1* allele, suggesting that these mutations might have caused the constitutive *MDR1* overexpression and the resulting multidrug resistance.

### *MDR1* Overexpression and Multidrug Resistance in Clinical C. albicans Isolates Are Caused by Gain-of-Function Mutations in *MRR1*

**Figure 5:**
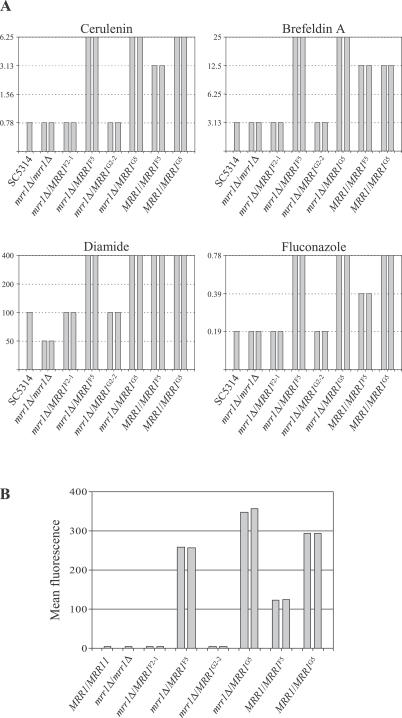
*MRR1* Gain-of-Function Alleles Cause Constitutive *MDR1* Overexpression and Multidrug Resistance (A) MICs (in μg ml^−1^) of the indicated metabolic inhibitors for the wild-type parental strain SC5314, two independently constructed homozygous *mrr1*Δ mutants, and derivatives carrying the indicated mutant *MRR1* alleles in the absence or presence of a wild-type *MRR1* allele. (B) *MDR1* promoter activity in a wild-type reporter strain, an *mrr1*Δ mutant, and derivatives carrying the indicated mutant *MRR1* alleles in the absence or presence of a wild-type *MRR1* allele. The strains were grown to log phase in YPD medium and the mean fluorescence of the cells was determined by flow cytometry.

To directly test whether the P683S and G997V mutations in Mrr1p are responsible for *MDR1* overexpression and drug resistance, we introduced the mutated *MRR1* alleles from isolates F5 and G5 as well as the corresponding wild-type alleles from isolates F2 and G2 into the *mrr1*Δ mutants of strain SC5314. All four *MRR1* alleles were integrated into one of the inactivated *mrr1*Δ alleles to ensure expression from the endogenous *MRR1* promoter (see Figure 2B and 2C, lanes 4–7). In each case, two independent correct transformants were used for further analysis. Figure 5A shows that, as noted above, deletion of *MRR1* in strain SC5314 did not affect its susceptibility to cerulenin, brefeldin A, and fluconazole, but the mutants displayed increased sensitivity to diamide, a compound that induces *MDR1* expression and is an Mdr1p substrate. Insertion of the *MRR1–1* allele from isolate F2 (which is identical to *MRR1* of strain SC5314, so this also represented a reinsertion of the original allele) or the *MRR1–2* allele from isolate G2 complemented the hypersusceptibility of the mutants to diamide, but did not increase resistance to cerulenin, brefeldin A, and fluconazole. In contrast, insertion of the mutated alleles from isolates F5 and G5 resulted in enhanced resistance to all compounds. To obtain direct evidence that the mutated *MRR1* alleles confer drug resistance by activating the *MDR1* promoter and, thus, mediate overexpression of the Mdr1p efflux pump, we integrated the various *MRR1* alleles into a derivative of the laboratory strain CAI4 that expresses the *GFP* reporter gene from the endogenous *MDR1* promoter. For this purpose, we first inactivated the *MRR1* wild-type alleles in the reporter strain CAG48B, as described above for the other strains, and then reinserted one of the four different *MRR1* alleles. Two independent transformants were kept for each *MRR1* allele. Figure 5B shows that, while the *MRR1* ^F2–1^ and *MRR1* ^G2–2^ alleles from the susceptible isolates had no effect, expression of the corresponding mutated *MRR1* ^F5^ and *MRR1* ^G5^ alleles resulted in strong *MDR1* promoter activity. *MDR1* upregulation by the mutated *MRR1* alleles was also independently confirmed by comparing *MDR1* mRNA levels in strains carrying wild-type or mutated *MRR1* alleles by quantitative real-time RT-PCR (see Figure S1B). These results demonstrated that the P683S and G997V mutations in Mrr1p caused constitutive *MDR1* overexpression and multidrug resistance.

### *MRR1* Gain-of-Function Alleles Mediate *MDR1* Overexpression and Multidrug Resistance in a Semi-Dominant Fashion

A mutation in the transcription factor *TAC1* has recently been shown to cause constitutive upregulation of the ABC-transporters *CDR1* and *CDR2* as well as drug resistance in certain C. albicans strains, but only after the strains had become homozygous for the mutated allele [28]. Since the resistant isolates F5 and G5 also had become homozygous for mutated *MRR1* alleles, we tested whether these alleles could mediate *MDR1* overexpression and drug resistance in the presence of a non-mutated, wild-type allele. Therefore, to produce strains that contained both a wild-type and a mutated allele, the *MRR1* alleles from isolates F5 and G5 were inserted into the inactivated *mrr1* allele in the heterozygous *MRR1*/*mrr1*Δ mutants derived from strain SC5314 (see Figure 2B and 2C, lanes 8 and 9). The mutated alleles conferred drug resistance also in the presence of a wild-type *MRR1* allele, although we observed a slightly reduced resistance as compared with the strains containing only a mutated *MRR1* allele, especially for the *MRR1* ^F5^ allele (see Figure 5A). To directly compare *MDR1* promoter activity in strains carrying only a mutated *MRR1* allele or both a mutated and a wild-type *MRR1* allele, the mutated *MRR1* alleles were also integrated into the inactivated *mrr1* allele of the heterozygous *MRR1*/*mrr1*Δ mutant with the P*_MDR1_-GFP* reporter fusion. Figure 5B shows that both mutated *MRR1* alleles were able to activate the *MDR1* promoter in the presence of a wild-type *MRR1* allele, but the degree of activation was lower than in strains containing only a mutated allele. Again, this effect was more pronounced for the *MRR1* ^F5^ allele. Taken together, these results demonstrate that the *MRR1* ^F5^ and *MRR1* ^G5^ alleles can act in a semi-dominant fashion and mediate *MDR1* overexpression and multidrug resistance in the presence of a nonmutated *MRR1* allele, but the presence of a wild-type *MRR1* allele reduces the activity of the *MRR1* alleles containing gain-of-function mutations.

### Identification of Mrr1p Target Genes

That deletion of *MRR1* from the drug-resistant C. albicans isolates F5 and G5 affected fluconazole resistance more strongly than deletion of *MDR1* suggests that Mrr1p controls the expression of additional genes that contribute to the increased fluconazole resistance of these isolates. Therefore, to identify the set of genes controlled by the transcription factor Mrr1p, we compared the gene expression profiles of isogenic strains expressing either a wild-type *MRR1* allele (*MRR1* ^F2–1^ or *MRR1* ^G2–2^) or its constitutively active, mutated counterpart (*MRR1* ^F5^ and *MRR1* ^G5^, respectively). In addition, we compared the gene expression profiles of the clinical isolates F5 and G5, which carry the gain-of-function *MRR1* alleles, with those of their *mrr1*Δ derivatives.

**Figure 6:**
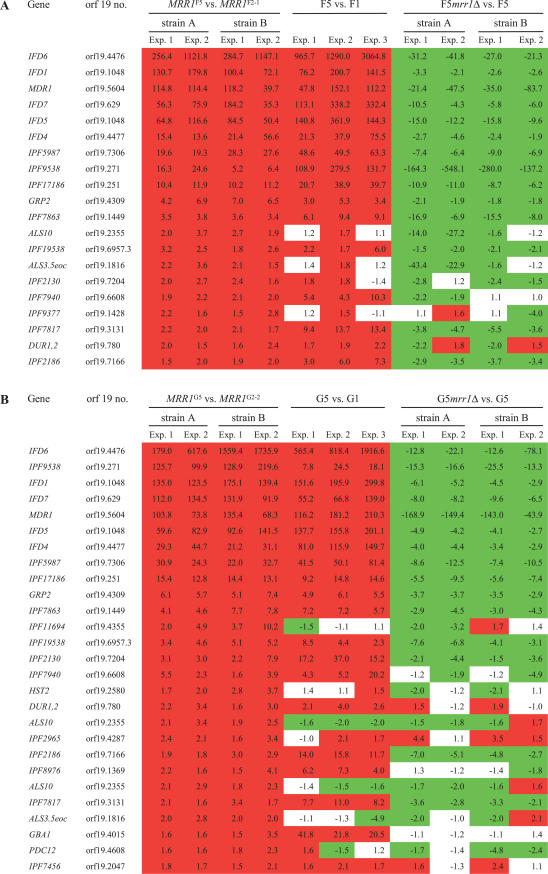
Identification of Mrr1p Target Genes by DNA Microarray Analysis (A) Genes that were upregulated by the P683S mutation in the SC5314 genetic background (*MRR1* ^F5^ versus *MRR1* ^F2–1^). The genes are ordered according to their average degree of upregulation in four repeat experiments performed with two independent transformants (strains A and B). The changes in the expression level of these genes in the drug-resistant clinical isolate F5 as compared with a matched susceptible isolate (F5 versus F1) and in two independently constructed *mrr1*Δ mutants (strains A and B) as compared with their wild-type progenitor F5 (F5*mrr1*Δ versus F5) are shown for comparison. (B) Genes that were upregulated by the G997V mutation in the SC5314 genetic background (*MRR1* ^G5^ versus *MRR1* ^G2–2^). The genes are ordered according to their average degree of upregulation in four repeat experiments performed with two independent transformants (strains A and B). The changes in the expression level of these genes in the drug-resistant clinical isolate G5 as compared with a matched susceptible isolate (G5 versus G1) and in two independently constructed *mrr1*Δ mutants (strains A and B) as compared with their wild-type progenitor G5 (*mrr1*Δ versus G5) are shown for comparison. The fold increase or decrease in the expression of the genes is given for each experiment. Upregulated genes are highlighted in red, downregulated genes are highlighted in green, and genes that were not differentially expressed are shown as white boxes in all pairwise comparisons.

As shown in Figure 6A and 6B, 20 and 27 genes were consistently upregulated in the transformants expressing the *MRR1* ^F5^ and *MRR1* ^G5^ alleles, respectively, and 19 of the 28 total genes were commonly upregulated by both gain-of-function alleles. As expected, *CDR1* and *CDR2* were not among the Mrr1p target genes. Strikingly, the 11 most strongly upregulated genes were the same in strains expressing the *MRR1* ^F5^ or the *MRR1* ^G5^ allele. All of these (plus three additional commonly upregulated genes) were also significantly overexpressed in the drug-resistant clinical isolates F5 and G5 as compared with the matched susceptible isolates F1 and G1, respectively, and downregulated again after deletion of *MRR1* from the resistant isolates F5 and G5. These 14 genes, therefore, represent a core set of genes whose expression is controlled by Mrr1p, and some of them might contribute to fluconazole resistance in the clinical isolates (see Discussion).

Twenty-eight genes (including *MRR1*) were consistently downregulated after deletion of *MRR1* in the resistant isolates F5 and G5 (see Table 1) A complete list of all genes that were found to be differentially regulated in the *mrr1*Δ mutants as compared with their wild-type parental strains is provided in Table S4. Fourteen of the 28 genes were also upregulated by the *MRR1* ^F5^ and *MRR1* ^G5^ alleles in the SC5314 background and in the resistant isolates F5 and G5 as compared with the matched susceptible isolates F1 and G1, respectively. The 14 genes were mainly those most strongly affected by the *MRR1* deletion. The other genes were either not consistently upregulated above the threshold level in the SC5314 transformants expressing only one mutated *MRR1* allele (e.g., *MRR1* itself) or possibly had a strain-specific dependence on *MRR1*.

**Table 1:**
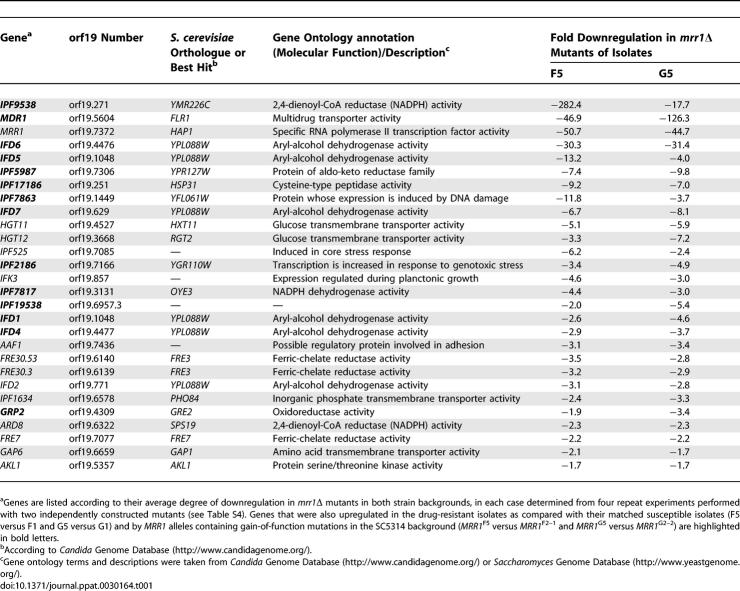
Genes Commonly Downregulated by *MRR1* Deletion in Isolates F5 and G5

Altogether, the results of the transcriptional profiling experiments provided a comprehensive list of genes that are regulated, directly or indirectly, by Mrr1p in various C. albicans strain backgrounds. The identification of these genes, in turn, provided clues about how gain-of-function mutations in *MRR1* contribute to fluconazole resistance of clinical C. albicans strains, besides causing overexpression of the *MDR1* efflux pump (see Discussion).

## Discussion

Since the initial report by Sanglard et al. more than ten years ago [3], many studies have shown that the major mechanism of resistance to the widely used antifungal agent fluconazole in clinical C. albicans isolates is the constitutive overexpression of efflux pumps, which actively transport this drug and other toxic substances out of the cell, thereby conferring multidrug resistance. It is well established that mutations in *trans*-regulatory factors are responsible for the upregulation of genes encoding efflux pumps in drug-resistant C. albicans isolates [24,32]; however, until recently the identity of these regulators has remained elusive. Two main factors, namely 1) the discovery of a *cis*-regulatory element in the promoters of *CDR1* and *CDR2* with features that are typical of binding sites of zinc cluster transcription factors, and 2) the observation that homozygosity at the mating type locus was linked with the development of azole resistance in certain clinical strains, led Coste et al. to exploit the available C. albicans genome sequence information to systematically search for candidate transcription factors that might regulate the expression of these efflux pumps. This strategy resulted in the identification of the zinc cluster transcription factor *TAC1*, which is located near the mating type locus, as the major regulator of *CDR1* and *CDR2* [27]. In contrast, no obvious criteria for a similar in silico search for a regulator of *MDR1*, the other efflux pump that mediates drug resistance in many clinical C. albicans isolates, were evident. Promoter deletion analyses performed by several research groups have identified three different activating regions in the *MDR1* promoter, two of which contain binding sites for the transcription factors Cap1p and Mcm1p [19,20,25,26]. However, each of these regions could be individually deleted from the full-length *MDR1* promoter without abrogating its constitutive activation in *MDR1* overexpressing C. albicans isolates, which suggested that a transcription factor other than Cap1p or Mcm1p causes the upregulation of *MDR1* in drug-resistant strains. Assuming that a common mechanism might be responsible for the constitutive *MDR1* upregulation in such strains, we compared the alterations in gene expression that occurred in three different *MDR1* overexpressing, drug-resistant C. albicans isolates. This approach led to the identification of *MRR1*, a zinc cluster transcription factor that was moderately upregulated in all three resistant isolates as compared with matched, drug-susceptible isolates, suggesting that this transcription factor might contribute to *MDR1* overexpression and/or drug resistance. A role of *MRR1* in fluconazole resistance would not have been easily inferred from genetic analysis of commonly used C. albicans laboratory strains, because deletion of *MRR1* from the model strain SC5314 did not result in hypersusceptibility of the mutants. However, the development of methods to inactivate genes in clinical C. albicans strains [21,30] allowed us to demonstrate the essential role of *MRR1* in drug resistance of two different *MDR1* overexpressing C. albicans isolates. Similar findings were previously obtained with the efflux pump *MDR1* itself, whose disruption in a C. albicans laboratory strain did not result in fluconazole hypersusceptibility, whereas *MDR1* inactivation in *MDR1* overexpressing strains reduced or abolished their resistance to fluconazole and other metabolic inhibitors [14,21,33]. We found that *MRR1* is not only responsible for the constitutive overexpression of *MDR1* in drug-resistant isolates, but also mediates the inducible *MDR1* expression in a drug-susceptible strain. The transcription factors Cap1p and Mcm1p have been implicated in the induction of *MDR1* expression by H_2_O_2_ and benomyl, respectively [20]. Interestingly, deletion of *MRR1* almost completely abolished the induction of the *MDR1* promoter in response to both of these stimuli, indicating that Mrr1p has a more central and essential role in the control of *MDR1* expression and, depending on the environmental conditions, may cooperate in different ways with these other transcription factors to regulate expression of the efflux pump.

The identification of *MRR1* as the central regulator of *MDR1* expression also enabled us to elucidate the genetic alterations that had occurred in drug-resistant C. albicans isolates and were responsible for *MDR1* overexpression and multidrug resistance. In two clinical isolates we found different single nucleotide substitutions in *MRR1* that resulted in amino acid exchanges in Mrr1p. The ability of the mutated *MRR1* alleles to activate the *MDR1* promoter and confer drug resistance when expressed in a C. albicans laboratory strain confirmed that these were indeed gain-of-function mutations that resulted in constitutive activation of the transcription factor. The two mutations, which were found in different regions of Mrr1p, most likely relieve the transcription factor from repression by an autoinhibitory domain or by another negatively acting factor that keeps Mrr1p in its inactive state in the absence of inducing signals. In both cases the *MDR1* overexpressing, drug-resistant isolate had become homozygous for the mutated *MRR1* allele, which suggested that Mrr1p containing a gain-of-function mutation would not be able to activate the *MDR1* promoter in the presence of wild-type Mrr1p. However, we found that both mutated *MRR1* alleles were able to induce *MDR1* expression and cause drug resistance in the presence of a wild-type allele, albeit at slightly reduced levels as compared with strains containing only the mutated allele. This indicates that the gain-of-function alleles acted in a semi-dominant fashion. The comparison of heterozygous and homozygous *mrr1*Δ mutants with their drug-resistant parental strains showed that the presence of two rather than only one mutated *MRR1* allele resulted in higher *MDR1* promoter activity and drug resistance. Therefore, the increased activity of the transcription factor in strains carrying two copies of a mutated *MRR1* allele instead of only one, coupled with a slight negative effect of wild-type Mrr1p on the activity of the activated form, appears to provide sufficient advantage during antimycotic therapy to select for the loss of heterozygosity once a gain-of-function mutation has occurred in one of the two *MRR1* alleles. Such a loss of heterozygosity readily occurs in C. albicans either by loss of one chromosome and duplication of the homologous chromosome or by mitotic recombination between the two homologous chromosomes, as has been well documented in several recent studies [28,34,35].

Interestingly, deletion of the transcription factor *MRR1* from *MDR1* overexpressing C. albicans isolates reduced fluconazole resistance of these strains even more than deletion of *MDR1* itself, suggesting that the gain-of-function mutations in *MRR1* contribute to fluconazole resistance of these strains by other mechanisms, in addition to causing overexpression of the efflux pump. We, therefore, aimed to find out which of the many alterations in gene expression seen in the drug-resistant C. albicans clinical isolates (see Figure 1 and Table S1) were caused by the *MRR1* mutations. When introduced into strain SC5314, both mutated *MRR1* alleles caused the upregulation of 19 genes including *MDR1* (note that some genes of the *IFD* family may in fact be alleles of the same gene, e.g., those that were originally designated as *IFD1* and *IFD5* in CandidaDB but have now been assigned the same orf19 name). Some additional genes were reproducibly upregulated by only one of the two mutated alleles (one for *MRR1* ^F5^ and eight for *MRR1* ^G5^), which could be explained by differential effects of the P683S and G997V mutations on Mrr1p activity at the respective target promoters. The expression of 14 of the 19 genes that were upregulated by both mutated *MRR1* alleles in the SC5314 background was found to be downregulated after deletion of *MRR1* in both drug-resistant clinical isolates F5 and G5. A considerable number of additional genes were affected by inactivation of *MRR1* in the clinical isolates (see Table S4), but only 28 genes were downregulated in the *mrr1*Δ mutants of both parental strains, indicating that the other effects of *MRR1* deletion depended on the strain background.

It is striking that of the core set of Mrr1p target genes (i.e., the 14 genes that were upregulated by the mutated *MRR1* alleles in the SC5314 background as well as upregulated in the drug-resistant isolates F5 and G5 and downregulated in the *mrr1*Δ mutants of these strains) many encode putative oxidoreductases (see Table 1). *IPF7817* is a member of the NAD(P)H oxidoreductase family that is strongly induced during oxidative stress [36,37]. It is supposed to be involved in the regulation of intracellular redox homeostasis, as mutants in which the gene was inactivated had increased intracellular levels of reactive oxygen species (ROS) and, presumably as a compensatory mechanism, upregulated other redox-related genes [38]. *IPF17186* is a member of the ThiJ/PfpI protein family [39]. Its ortholog in S. cerevisiae, also named *HSP31*, is induced by the transcription factor Yap1p in response to oxidative stress. Also, an *hsp31*Δ mutant is hypersensitive to a subset of ROS generators, suggesting that Hsp31p may protect the cell against oxidative stress [40]. *GRP2* is a homolog of *S. cerevisiae GRE2*, which encodes a stress-induced NADH-dependent methylglyoxal reductase that is regulated by the pleiotropic drug resistance regulator Pdr1p. A *gre2*Δ mutant exhibited a growth defect under conditions of membrane stress and activated *ERG* genes as a compensatory mechanism, and it displayed an increased sensitivity to ergosterol biosynthesis inhibitors [41]. *IFD1*, *IFD4*, *IFD5*, *IFD6*, and *IFD7* encode proteins of the aldo-keto reductase family and are homologs of the putative aryl-alcohol dehydrogenase *YPL088w* of S. cerevisiae. Interestingly, *YPL088w* is regulated by the transcriptional regulators Yrr1p and Yrm1p, which are involved in the control of multidrug resistance [42]. Similarly, *IPF5987* encodes a member of the aldo-keto reductase family, and its ortholog in S. cerevisiae is also transcriptionally regulated by Yrr1p and Yrm1p [42].

*MDR1* and other Mrr1p target genes, almost all of which have also been found to be upregulated in another *MDR1* overexpressing clinical C. albicans isolate [18], are induced in the presence of chemicals that exert oxidative stress upon the cells, like hydrogen peroxide or diamide. They are also induced by the microtubule destabilizing agent benomyl [16,18–20]. Interestingly, benomyl treatment causes lipid peroxidation and glutathione depletion in rats and these effects were blocked by treatment with antioxidants, suggesting that the in vivo toxicity of benomyl may be associated with oxidative stress to cellular membranes [43]. Therefore, it seems possible that the activation of Mrr1p by all these compounds may be a response to oxidative damage of the cells, and the function of many of the target genes that are induced by activated Mrr1p could be to restore the intracellular redox balance. This may also explain how the upregulation of these genes contributes to fluconazole resistance of clinical C. albicans strains containing *MRR1* gain-of-function mutations. In addition to inhibiting ergosterol biosynthesis, azoles have been shown to increase the level of endogenous reactive oxygen species in C. albicans cells, and the decrease in cell viability associated with miconazole treatment was significantly prevented by addition of an antioxidant [44]. These observations suggest that ROS plays a role in the mechanism of action of azole antifungal agents and that ROS detoxification mechanisms may contribute to azole resistance. In contrast to other compounds that cause oxidative stress, like hydrogen peroxide or diamide, fluconazole does not induce *MDR1* expression [8,16]. Therefore, the constitutive upregulation of *MDR1* and other Mrr1p target genes in strains containing *MRR1* gain-of-function mutations provides protection of the cells against this antifungal agent.

A major regulator of the oxidative stress response in C. albicans is the bZIP transcription factor Cap1p [36,45,46]. Like *MDR1*, several other Mrr1p target genes (*IPF7817*, *IPF17186*, and *GRP2*) contain a YRE element, the putative binding site for Cap1p, in their promoters. Their expression is induced by hydrogen peroxide in a Cap1p-dependent manner [18,20,36,45]. Therefore, Cap1p and Mrr1p may act together to regulate expression of these genes in response to oxidative stress. Cap1p also controls the expression of other genes that are involved in the oxidative stress response, like the thioredoxin reductase *TRR1*, the glutathione reductase *GLR1*, the glutathione *S*-transferase *GTT1*, and the superoxide dismutase *SOD2* [36,45], and which were not found among the Mrr1p target genes. Unlike *CAP1* inactivation [45], *MRR1* deletion or *MRR1* gain-of-function mutations had no effect on the susceptibility of C. albicans to H_2_O_2_ (unpublished data), which in contrast to diamide is not a substrate of the Mdr1p efflux pump [23]. Therefore, the Cap1p target genes that are not controlled by Mrr1p and that are typical oxidative stress-response genes seem to be more important for the resistance of C. albicans to hydrogen peroxide, while the Mrr1p target genes contribute to fluconazole resistance, presumably because the two compounds cause different types of damage within the cells. The precise function of most of the Mrr1p target genes is currently unknown and their potential involvement in an oxidative stress response remains speculative. Alternatively, it is possible that fluconazole treatment causes the accumulation of other toxic molecules that are eliminated by the combined action of the oxidoreductases and other gene products whose expression is regulated by Mrr1p.

The identification of *MRR1* as the major regulator of *MDR1* expression and the elucidation of the mutations in clinical isolates that cause constitutive activity of this transcription factor represent a major step forward in our understanding of multidrug resistance development in C. albicans. Important questions that can now be addressed include how Mrr1p is normally activated in response to inducing signals, how gain-of-function mutations cause constitutive activation of the transcription factor, and how Mrr1p interacts with other putative *MDR1* regulators like Cap1p and Mcm1p to control expression of its target genes.

## Materials and Methods

### Strains and growth conditions.

C. albicans strains used in this study are listed in the supporting Table S2. All strains were stored as frozen stocks with 15% glycerol at −80 °C. The strains were routinely grown in YPD medium (10 g yeast extract, 20 g peptone, 20 g glucose per liter) at 30 °C. To prepare solid media, 1.5% agar was added before autoclaving. For induction of the *MDR1* promoter with benomyl or H_2_O_2_, overnight cultures of reporter strains were diluted 10^−2^ in three flasks with fresh YPD medium and grown for 3 h. Fifty μg/ml of benomyl or 0.005% H_2_O_2_ was then added to one of the cultures and the cells were grown for an additional hour. The fluorescence of the cells was quantified by FACS analysis.

### Plasmid constructions.

The coding region of the *MRR1* gene of C. albicans strain SC5314 was amplified by PCR with the primers ZCF36–1 and ZCF36–2 (primer sequences are given in Table S5). The PCR product was digested at the introduced *Sal*I and *Bgl*II restriction sites and substituted for the *OPT4* open reading frame (ORF) in the *Xho*I/*Bgl*II-digested pOPT4E1 [47] to generate pZCF36E1. The sequence of the cloned *MRR1* gene was identical to that found in the genome sequence of C. albicans strain SC5314 (orf19.7372). An *MRR1* deletion construct was generated in the following way: A *Sac*I-*Sac*II fragment containing *MRR1* upstream sequences from positions −314 to +15 with respect to the start codon was amplified with the primers ZCF36–3 and ZCF36–4, and an *Xho*I-*Apa*I fragment containing *MRR1* downstream sequences from positions +3260 to +3557 was amplified with the primers ZCF36–5 and ZCF36–6. The *MRR1* upstream and downstream fragments were cloned on both sides of the *SAT1* flipper cassette in plasmid pSFS1 [30] to result in pZCF36M2 in which the *MRR1* coding region from positions +16 to +3259 (66 bp before the stop codon) is replaced by the *SAT1* flipper (see Figure 2A). DNA fragments containing the N-terminal part and upstream sequences of the *MRR1* alleles of the clinical C. albicans isolates F2, F5, G2, and G5 were amplified with the primers ZCF36–3 and ZCF36seq6, digested at the introduced *Sac*I site and at an internal *Eco*RI site, and cloned in pBluescript to generate pZCF36NF2A and D, pZCF36NF5A, pZCF36NG2B and D, and pZCF36G5A, respectively. DNA fragments containing the *MRR1* C-terminal part and downstream sequences were amplified with the primers ZCF36–1 and ZCF36–6, digested at the internal *Eco*RI site and at the introduced *Sac*I site, and cloned in pBluescript to obtain pZCF36CF2B and C, pZCF36CF5C, pZCF36CG2A and D, and pZCF36CG5A, respectively. The complete ORFs and upstream sequences of the *MRR1–1* alleles of isolates F2 and G2 were also amplified with the primers ZCF36–3 and ZCF36–8 and cloned in pBluescript to yield pZCF36TF2–1 and pZCF36TG2–1, respectively. To express wild-type and mutated *MRR1* alleles in *mrr1*Δ mutants, an *Eco*RI-*Sal*I fragment from pZCF36E1 containing the C-terminal part of *MRR1*, the *ACT1* transcription termination sequence, and part of the *caSAT1* selection marker was cloned into the *Eco*RI/*Sal*I-digested pZCF36NF2A to obtain pZCF36K1. An *MRR1* downstream fragment was then amplified from SC5314 genomic DNA with the primers ZCF36–7 and ZCF36–6, digested at the introduced *Nsi*I and *Apa*I sites, and cloned together with a *Bgl*II-*Pst*I fragment from pZCF36E1 containing the *ACT1* transcription termination sequence and the *caSAT1* selection marker into the *Bgl*II/*Apa*I-digested pZCF36K1 to generate pZCF36K2, which contains the *MRR1–1* allele of isolate F2 (which is identical to the *MRR1* gene of strain SC5314). Plasmid pZCF36K3, which contains the mutated *MRR1* allele from isolate F5, was obtained by substituting an *Eco*RI-*Pst*I fragment from pZCF36CF5C for the corresponding fragment in pZCF36K2. For expression of the *MRR1–2* allele of isolate G2, an *Nde*I-*Eco*RI fragment from pZCF36NG2B and an *Eco*RI-*Pst*I fragment from pZCF36CG2D were substituted for the corresponding region in pZCF36K2, resulting in pZCF36K4. Replacement of the *Eco*RI-*Pst*I fragment in this plasmid by the corresponding region from pZCF36CG5A generated pZCF36K5, which contains the mutated *MRR1* allele from isolate G5. Plasmid pMPG2S, which contains a P*_MDR1_-GFP* reporter fusion, was constructed by substituting the *caSAT1* selection marker from pSAT1 [30] for the *URA3* marker in the previously described plasmid pMPG2 [25].

### C. albicans transformation.

C. albicans strains were transformed by electroporation [48] with gel-purified inserts from the plasmids described above. Nourseothricin-resistant transformants were selected on YPD agar plates containing 200 μg/ml nourseothricin (Werner Bioagents) as described previously [30]. The correct genomic integration of all constructs was confirmed by Southern hybridization.

### Isolation of genomic DNA and Southern hybridization.

Genomic DNA from C. albicans was isolated as described previously [49]. 10 μg of DNA was digested with appropriate restriction enzymes, separated on a 1% agarose gel and, after ethidium bromide staining, transferred by vacuum blotting onto a nylon membrane and fixed by UV cross-linking. Southern hybridization with enhanced chemiluminescence-labeled probes was performed with the Amersham ECL^TM^ Direct Nucleic Acid Labeling and Detection System (GE Healthcare) according to the instructions of the manufacturer.

### Drug susceptibility tests.

Stock solutions of the drugs were prepared as follows. Fluconazole (1 mg/ml) and diamide (20 mg/ml) were dissolved in water, while cerulenin (5 mg/ml) and brefeldin A (5 mg/ml) were dissolved in DMSO. In the assays, serial 2-fold dilutions in the assay medium were prepared from the following initial concentrations: cerulenin, 200 μg/ml; brefeldin A, 200 μg/ml; diamide, 800 μg/ml; fluconazole, 200 μg/ml. Susceptibility tests were carried out in high resolution medium (14.67 g HR-Medium [Oxoid GmbH], 1 g NaHCO_3_, 0.2 M phosphate buffer [pH 7.2]), using a previously described microdilution method [50]. Readings were done after 48 h.

### FACS analysis.

Fluorescence-activated cell sorter (FACS) analysis was performed with a FACSCalibur cytometry system equipped with an argon laser emitting at 488 nm (Becton Dickinson). Fluorescence was measured on the FL1 fluorescence channel equipped with a 530-nm band-pass filter. Twenty thousand cells were analyzed per sample and were counted at low flow rate. Fluorescence and forward scatter data were collected by using logarithmic amplifiers. The mean fluorescence values were determined with CellQuest Pro (Becton Dickinson) software.

### DNA microarray analysis.

The nucleotide sequences corresponding to 6,165 ORFs for C. albicans were downloaded from the Galar Fungail European Consortium (Assembly 6, http://www.pasteur.fr/Galar_Fungail/CandidaDB). Following the Affymetrix Design Guide, we designed two separate probe sets for each ORF, each consisting of 13 perfect match and 13 mismatch overlapping 25 bp oligonucleotides, to the 3′ 600 bp region. For ORFs less than 600 bp in length, the sequence was divided in two equal segments for subsequent design procedures. For quality control and normalization purposes, we made 2–3 additional probe sets spanning the entire sequence of the C. albicans 18S rRNA (GenBank Accession M60302), genes encoding GAPDH, actin and Mdr1p (Bmr1p) in addition to the standard Affymetrix controls (BioB, C, D, cre, DAP, PHE, LYS, THR). The probe selection was performed by the Chip Design group at Affymetrix, Inc. using their proprietary algorithm to calculate probe set scores, which includes a probe quality metric, cross-hybridization penalty, and gap penalty. The probe sets were then examined for cross-hybridization against all other sequences in the C. albicans genome as well as a number of constitutively expressed genes and rRNA from other common organisms. Consequently, for some target regions we were not able to design high quality probe sets. In the end, the GeneChip contained 10,736 probe sets including 9 controls, 6,123 unique ORFs, and duplicate probe sets for 4,604 ORFs. The duplicate probe sets are made to distinct regions of the ORF, thereby allowing 2 independent measurements of the mRNA level for that particular gene. The C. albicans custom Affymetrix NimbleExpress Arrays (CAN04a530004N) were manufactured by NimbleGen Systems [51] per our specification.

### RNA preparation for microarrays.

Total RNA was isolated using the hot SDS-phenol method [52]. Frozen cells were suspended in 12 ml of 50 mM sodium acetate (pH 5.2), 10 mM EDTA at room temperature, after which 1 ml of 20% sodium dodecyl sulphate and 12 ml of acid phenol (Fisher Scientific) were added. This mixture was incubated 10 min at 65 °C with mixing each minute, cooled on ice for 5 min, and centrifuged for 15 min at 12,000*g*. Supernatants were transferred to new tubes containing 15 ml of chloroform, mixed, and centrifuged at 200*g* for 10 min. The aqueous layer was removed to new tubes, RNA was precipitated with 1 vol isopropanol and 0.1 vol 2 M sodium acetate (pH 5.0), and then collected by centrifugation at 17,000*g* for 35 min at 4 °C. The RNA pellet was suspended in 10 ml of 70% ethanol, collected again by centrifugation, and suspended in nuclease-free water.

### cRNA synthesis and labeling.

Immediately prior to cDNA synthesis, the purity and concentration of RNA samples were determined from A_260_/A_280_ readings, and RNA integrity was determined by capillary electrophoresis using the RNA 6000 Nano Laboratory-on-a-Chip kit and Bioanalyzer 2100 (Agilent Technologies) as per the manufacturer's instructions. First and second strand cDNA was synthesized from 15 μg total RNA using the SuperScript Double-Stranded cDNA Synthesis Kit (Invitrogen) and oligo-dT24-T7 primer (PrOligo) according to the manufacturer's instructions. cRNA was synthesized and labeled with biotinylated UTP and CTP by in vitro transcription using the T7 promoter-coupled double-stranded cDNA as template and the Bioarray HighYield RNA Transcript Labeling Kit (ENZO Diagnostics). Double-stranded cDNA synthesized from the previous steps was washed twice with 70% ethanol and suspended in 22 μl of RNase-free water. The cDNA was incubated as recommended with reaction buffer, biotin-labeled ribonucleotides, dithtiothreitol, RNase inhibitor mix, and T7 RNA polymerase for 5 h at 37 °C. The labeled cRNA was separated from unincorporated ribonucleotides by passing through a CHROMA SPIN-100 column (Clontech) and ethanol precipitated at −20 °C overnight.

### Oligonucleotide array hybridization and analysis.

The cRNA pellet was suspended in 10 μl of RNase-free water and 10 μg was fragmented by ion-mediated hydrolysis at 95 °C for 35 min in 200 mM Tris-acetate (pH 8.1), 500 mM potassium acetate, 150 mM magnesium acetate. The fragmented cRNA was hybridized for 16 h at 45 °C to the C. albicans NimbleExpress GeneChip arrays. Arrays were washed at 25 °C with 6 × SSPE, 0.01% Tween 20 followed by a stringent wash at 50 °C with 100 mM MES, 0.1 M NaCl, 0.01% Tween 20. Hybridizations and washes employed the Affymetrix Fluidics Station 450 using their standard EukGE-WS2v5 protocol. The arrays were then stained with phycoerythrein-conjugated streptavidin (Molecular Probes) and the fluorescence intensities were determined using the GCS 3000 high-resolution confocal laser scanner (Affymetrix). The scanned images were analysed using software resident in GeneChip Operating System v2.0 (Affymetrix). Sample loading and variations in staining were standardized by scaling the average of the fluorescent intensities of all genes on an array to a constant target intensity (250). The signal intensity for each gene was calculated as the average intensity difference, represented by [Σ(PM − MM)/(number of probe pairs)], where PM and MM denote perfect-match and mismatch probes.

### Microarray data analysis.

The scaled gene expression values from GeneChip Operating System v2.0 software were imported into GeneSpring 7.2 software (Agilent Technologies) for preprocessing and data analysis. Probe sets were deleted from subsequent analysis if they were called absent by the Affymetrix criterion and displayed an absolute value below 20 in all experiments. The expression value of each gene was normalized to the median expression of all genes in each chip as well as the median expression for that gene across all chips in the study. Pairwise comparison of gene expression was performed for each matched experiment. Among direct comparisons between matched clinical isolates, genes were considered to be differentially expressed if their change in expression was ≥ 1.5-fold in three independent experiments.

### Quantitative real-time RT-PCR.

An aliquot of the RNA preparations from the samples used in the microarray experiments was saved for quantitative real-time RT-PCR follow-up studies. First-strand cDNAs were synthesized from 2 μg of total RNA in a 21-μl reaction volume using the SuperScript First-Strand Synthesis System for RT-PCR (Invitrogen) in accordance with the manufacturer's instructions. Quantitative real-time PCRs were performed in triplicate using the 7000 Sequence Detection System (Applied Biosystems). Independent PCRs were performed using the same cDNA for both the gene of interest and the 18S rRNA, using the SYBR Green PCR Master Mix (Applied Biosystems). Gene-specific primers were designed for the gene of interest and the 18S rRNA using Primer Express software (Applied Biosystems) and the Oligo Analysis & Plotting Tool (QIAGEN) and are shown in Table S5. The PCR conditions consisted of AmpliTaq Gold activation at 95 °C for 10 min, followed by 40 cycles of denaturation at 95 °C for 15 s and annealing/extension at 60 °C for 1 min. A dissociation curve was generated at the end of each PCR cycle to verify that a single product was amplified using software provided with the 7000 Sequence Detection System. The change in fluorescence of SYBR Green I dye in every cycle was monitored by the system software, and the threshold cycle (C*_T_*) above the background for each reaction was calculated. The C*_T_* value of 18S rRNA was subtracted from that of the gene of interest to obtain a ΔC*_T_* value. The ΔC*_T_* value of an arbitrary calibrator (e.g., untreated sample) was subtracted from the ΔC*_T_* value of each sample to obtain a ΔΔC*_T_* value. The gene expression level relative to the calibrator was expressed as 2^−ΔΔCT^.

## Supporting Information

Figure S1 Analysis of *MDR1* Expression in Clinical C. albicans Isolates, *mrr1*Δ Mutants, and Strains Expressing *MRR1* Gain-of-Function Alleles by Quantitative Real-Time RT-PCR (90 KB PDF)

Table S1 Genes that Were Differentially Expressed in the Flucoazole-Resistant Isolates F5, G5, and 6692 as Compared with the Matched Fluconazole-Susceptible Isolates F1, G1, and 5833, Respectively Sheet 1 shows all upregulated genes; genes that were upregulated in two or all three drug-resistant isolates are highlighted in light orange and dark orange, respectively. Sheet 2 shows all downregulated genes; genes that were downregulated in two or all three drug-resistant isolates are highlighted in light green and dark green, respectively.
(510 KB XLS)

Table S2 C. albicans Strains Used in This Study (137 KB DOC)

Table S3 Allelic Differences in the *MRR1* Alleles of Isolates F2, F5, G2, and G5 (166 KB DOC)

Table S4 Genes that Were Differentially Expressed in the *mrr1*Δ Mutants of the Drug-Resistant Isolates F5 and G5 as Compared with Their Parental Strains Sheet 1 shows all downregulated genes; sheet 2 shows all upregulated genes. Genes that were downregulated or upregulated in *mrr1*Δ mutants of both F5 and G5 are highlighted in green and orange, respectively.
(260 KB XLS)

Table S5 Primers Used in This Study (40 KB DOC)

### Accession Numbers

The coding sequences of the *MRR1* alleles described in this study have been deposited in GenBank (http://www.ncbi.nlm.nih.gov/Genbank/index.html) with the following accession numbers: EU139261 (*MRR1* ^F2–1^), EU139262 (*MRR1* ^F2–2^), EU139263 (*MRR1* ^F5^), EU139264 (*MRR1* ^G2–1^), EU139265 (*MRR1* ^G2–2^), EU139266 (*MRR1* ^G5^).

## Abbreviations

ABC: ATP-binding cassette
C_*T*_: threshold cycle
FACS: fluorescence-activated cell sorter
GFP: green fluorescent protein
*MDR1*: multidrug resistance
*MRR1*: multidrug resistance regulator
ORF: open reading frame
PCR: polymerase chain reaction
ROS: reactive oxygen species
RT: reverse transcription
