# Supplementary material for: The Transcription Factor Mrr1p Controls Expression of the MDR1 Efflux Pump and Mediates Multidrug Resistance in Candida albicans
Source: PLoS Pathog. 2007 Nov 2;3(11):e164. doi: 10.1371/journal.ppat.0030164 (PMC2048531; doi:10.1371/journal.ppat.0030164)
Supplement: Supplementary file 1 [file ppat.0030164.sg001.pdf]

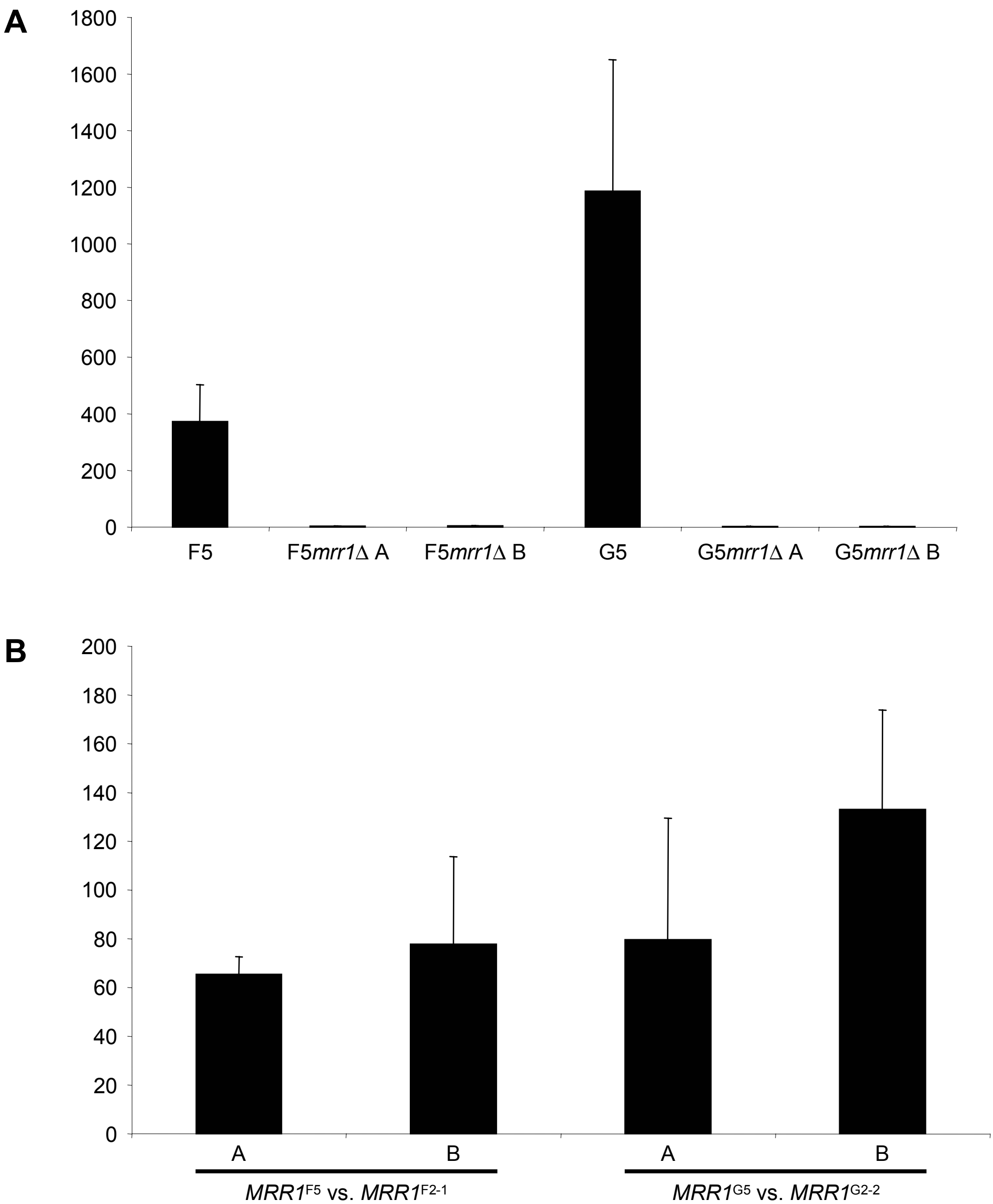

Figure S1. Analysis of *MDR1* expression by quantitative real-time RT-PCR

(A) Relative fold change in *MDR1* expression in the fluconazole-resistant isolates F5 and G5 and two independently derived *mrr1*Δ mutants of each parental strain as compared with the matched fluconazole-susceptible isolates F1 and G1, respectively. Shown are the means and standard errors from three experiments.

(B) Relative fold change in *MDR1* expression in strains expressing mutated *MRR1* alleles (*MRR1*^{F5} and *MRR1*^{G5}) as compared with strains expressing the corresponding non-mutated alleles (*MRR1*$^{F2\text{-}1}$ and *MRR1*$^{G2\text{-}2}$, respectively). Shown are the means and standard errors from three experiments performed with two independently constructed strains in each case.
