# Supplementary material for: The Transcription Factor Mrr1p Controls Expression of the MDR1 Efflux Pump and Mediates Multidrug Resistance in Candida albicans
Source: PLoS Pathog. 2007 Nov 2;3(11):e164. doi: 10.1371/journal.ppat.0030164 (PMC2048531; doi:10.1371/journal.ppat.0030164)
Supplement: Supplementary file 2 [file ppat.0030164.st002.doc]

**Table S2: *C. albicans*** strains used in this study

| Strain | Parent | Relevant characteristics or genotype | Reference |
| --- | --- | --- | --- |
| Wild-type parental strains and clinical isolates | | | |
| SC5314 |  | Wild-type *C. albicans* model strain | [1] |
| CAI4 | SC5314 | *ura3*Δ::*imm434/ura3*Δ::*imm434* | [2] |
| F1 |  | Clinical isolate from patient F, fluconazole-susceptible | [3] |
| F2 |  | Clinical isolate from patient F, fluconazole-susceptible | [3] |
| F5 |  | Clinical isolate from patient F, fluconazole-resistant | [3] |
| G1 |  | Clinical isolate from patient G, fluconazole-susceptible | [3] |
| G2 |  | Clinical isolate from patient G, fluconazole-susceptible | [3] |
| G5 |  | Clinical isolate from patient G, fluconazole-resistant | [3] |
| 5833 |  | Clinical isolate, fluconazole-susceptible | [4] |
| 6692 |  | Clinical isolate, fluconazole-resistant | [4] |
|  |  |  |  |
| *mdr1*Δ mutants | | | |
| F5M432 | F5 | *mdr1*Δ::*FRT*/*mdr1*Δ::*FRT* | [5] |
| G5M432 | G5 | *mdr1*Δ::*FRT*/*mdr1*Δ::*FRT* | [5] |
|  |  |  |  |
| *mrr1*Δ mutants | | | |
| SCMRR1M1A and -B | SC5314 | *MRR1*/*mrr1*Δ::*SAT1-FLIP* ^a^ | this study |
| SCMRR1M2A | SCMRR1M1A | *MRR1*/*mrr1*Δ::*FRT* | this study |
| SCMRR1M2B | SCMRR1M1B | *MRR1*/*mrr1*Δ::*FRT* | this study |
| SCMRR1M3A | SCMRR1M2A | *mrr1*Δ::*SAT1-FLIP*/*mrr1*Δ::*FRT* | this study |
| SCMRR1M3B | SCMRR1M2B | *mrr1*Δ::*SAT1-FLIP*/*mrr1*Δ::*FRT* | this study |
| SCMRR1M4A | SCMRR1M3A | *mrr1*Δ::*FRT*/*mrr1*Δ::*FRT* | this study |
| SCMRR1M4B | SCMRR1M3B | *mrr1*Δ::*FRT*/*mrr1*Δ::*FRT* | this study |
|  |  |  |  |
| F5MRR1M1A and -B | F5 | *MRR1*/*mrr1*Δ::*SAT1-FLIP* | this study |
| F5MRR1M2A | F5MRR1M1A | *MRR1*/*mrr1*Δ::*FRT* | this study |
| F5MRR1M2B | F5MRR1M1B | *MRR1*/*mrr1*Δ::*FRT* | this study |
| F5MRR1M3A | F5MRR1M2A | *mrr1*Δ::*SAT1-FLIP*/*mrr1*Δ::*FRT* | this study |
| F5MRR1M3B | F5MRR1M2B | *mrr1*Δ::*SAT1-FLIP*/*mrr1*Δ::*FRT* | this study |
| F5MRR1M4A | F5MRR1M3A | *mrr1*Δ::*FRT*/*mrr1*Δ::*FRT* | this study |
| F5MRR1M4B | F5MRR1M3B | *mrr1*Δ::*FRT*/*mrr1*Δ::*FRT* | this study |
|  |  |  |  |
| G5MRR1M1A and -B | G5 | *MRR1*/*mrr1*Δ::*SAT1-FLIP* | this study |
| G5MRR1M2A | G5MRR1M1A | *MRR1*/*mrr1*Δ::*FRT* | this study |
| G5MRR1M2B | G5MRR1M1B | *MRR1*/*mrr1*Δ::*FRT* | this study |
| G5MRR1M3A | G5MRR1M2A | *mrr1*Δ::*SAT1-FLIP*/*mrr1*Δ::*FRT* | this study |
| G5MRR1M3B | G5MRR1M2B | *mrr1*Δ::*SAT1-FLIP*/*mrr1*Δ::*FRT* | this study |
| G5MRR1M4A | G5MRR1M3A | *mrr1*Δ::*FRT*/*mrr1*Δ::*FRT* | this study |
| G5MRR1M4B | G5MRR1M3B | *mrr1*Δ::*FRT*/*mrr1*Δ::*FRT* | this study |
|  |  |  |  |
| Strains containing a P*_MDR1_-GFP* reporter fusion | | | |
| SCMPG2A and -B | SC5314 | *MRR1*/*MRR1*  *ACT1*/*act1*::P*_MDR1_-GFP-caSAT1* | this study |
| SCMRR1M2MPG2A | SCMRR1M2A | *MRR1*/*mrr1*Δ::*FRT*  *ACT1*/*act1*::P*_MDR1_-GFP-caSAT1* | this study |
| SCMRR1M2MPG2B | SCMRR1M2B | *MRR1*/*mrr1*Δ::*FRT*  *ACT1*/*act1*::P*_MDR1_-GFP-caSAT1* | this study |
| SCMΡΡ1M4MPG2A | SCMRR1M4A | *mrr1*Δ::*FRT*/*mrr1*Δ::*FRT*  *ACT1*/*act1*::P*_MDR1_-GFP-caSAT1* | this study |
| SCMRR1M4MPG2B | SCMRR1M4B | *mrr1*Δ::*FRT*/*mrr1*Δ::*FRT*  *ACT1*/*act1*::P*_MDR1_-GFP-caSAT1* | this study |
| F5MPG2A and -B | F5 | *MRR1*/*MRR1*  *ACT1*/*act1*::P*_MDR1_-GFP-caSAT1* | this study |
| F5MRR1M2MPG2A | F5MRR1M2A | *MRR1*/*mrr1*Δ::*FRT*  *ACT1*/*act1*::P*_MDR1_-GFP-caSAT1* | this study |
| F5MRR1M2MPG2B | F5MRR1M2B | *MRR1*/*mrr1*Δ::*FRT*  *ACT1*/*act1*::P*_MDR1_-GFP-caSAT1* | this study |
| F5MRR1M4MPG2A | F5MRR1M4A | *mrr1*Δ::*FRT*/*mrr1*Δ::*FRT*  *ACT1*/*act1*::P*_MDR1_-GFP-caSAT1* | this study |
| F5MRR1M4MPG2B | F5MRR1M4B | *mrr1*Δ::*FRT*/*mrr1*Δ::*FRT*  *ACT1*/*act1*::P*_MDR1_-GFP-caSAT1* | this study |
| G5MPG2A and -B | G5 | *MRR1*/*MRR1*  *ACT1*/*act1*::P*_MDR1_-GFP-caSAT1* | this study |
| G5MRR1M2MPG2A | G5MRR1M2A | *MRR1*/*mrr1*Δ::*FRT*  *ACT1*/*act1*::P*_MDR1_-GFP-caSAT1* | this study |
| G5MRR1M2MPG2B | G5MRR1M2B | *MRR1*/*mrr1*Δ::*FRT*  *ACT1*/*act1*::P*_MDR1_-GFP-caSAT1* | this study |
| G5MRR1M4MPG2A | G5MRR1M4A | *mrr1*Δ::*FRT*/*mrr1*Δ::*FRT*  *ACT1*/*act1*::P*_MDR1_-GFP-caSAT1* | this study |
| G5MRR1M4MPG2B | G5MRR1M4B | *mrr1*Δ::*FRT*/*mrr1*Δ::*FRT*  *ACT1*/*act1*::P*_MDR1_-GFP-caSAT1* | this study |
| CAG48B | CAI4 | *MDR1*/*mdr1*::P*_MDR1_-GFP-URA3*  *MRR1*/*MRR1* | J. Morschhäuser, unpublished |
| CAG48MRR1M1B | CAG48B | *MDR1*/*mdr1*::P*_MDR1_-GFP-URA3*  *MRR1*/*mrr1*Δ::*SAT1-FLIP* | this study |
| CAG48MRR1M2B | CAG48MRR1M1B | *MDR1*/*mdr1*::P*_MDR1_-GFP-URA3*  *MRR1*/*mrr1*Δ::*FRT* | this study |
| CAG48MRR1M3B | CAG48MRR1M2B | *MDR1*/*mdr1*::P*_MDR1_-GFP-URA3*  *mrr1*Δ::*SAT1-FLIP*/*mrr1*Δ::*FRT* | this study |
| CAG48MRR1M4B | CAG48MRR1M3B | *MDR1*/*mdr1*::P*_MDR1_-GFP-URA3*  *mrr1*Δ::*FRT*/*mrr1*Δ::*FRT* | this study |
| CAG48MRR1E1B | CAG48B | *MDR1*/*mdr1*::P*_MDR1_-GFP-URA3*  *ADH1*/*adh1*::P*_ADH1_-MRR1-caSAT1* | this study |
| CAG48ADH1B | CAG48B | *MDR1*/*mdr1*::P*_MDR1_-GFP-URA3*  *ADH1*/*adh1*::*caSAT1* | this study |
|  |  |  |  |
| *mrr1*Δ mutants containing a reintegrated wild-type or mutated *MRR1* allele | | | |
| SCMRR1M4K2A | SCMRR1M4A | *mrr1*Δ/*MRR1*^F2-1^*-caSAT1* | this study |
| SCMRR1M4K2B | SCMRR1M4B | *mrr1*Δ/*MRR1*^F2-1^*-caSAT1* | this study |
| SCMRR1M4K3A | SCMRR1M4A | *mrr1*Δ/*MRR1*^F5^*-caSAT1* | this study |
| SCMRR1M4K3B | SCMRR1M4B | *mrr1*Δ/*MRR1*^F5^*-caSAT1* | this study |
| SCMRR1M4K4A | SCMRR1M4A | *mrr1*Δ/*MRR1*^G2-2^*-caSAT1* | this study |
| SCMRR1M4K4B | SCMRR1M4B | *mrr1*Δ/*MRR1*^G2-2^*-caSAT1* | this study |
| SCMRR1M4K5A | SCMRR1M4A | *mrr1*Δ/*MRR1*^G5^*-caSAT1* | this study |
| SCMRR1M4K5B | SCMRR1M4B | *mrr1*Δ/*MRR1*^G5^*-caSAT1* | this study |
| SCMRR1M2K3A | SCMRR1M2A | *MRR1*/*MRR1*^F5^*-caSAT1* | this study |
| SCMRR1M2K3B | SCMRR1M2B | *MRR1*/*MRR1*^F5^*-caSAT1* | this study |
| SCMRR1M2K5A | SCMRR1M2A | *MRR1*/*MRR1*^G5^*-caSAT1* | this study |
| SCMRR1M2K5B | SCMRR1M2B | *MRR1*/*MRR1*^G5^*-caSAT1* | this study |
| CAG48MRR1M4K2B1 and ‑2 | CAG48MRR1M4B | *MDR1*/*mdr1*::P*_MDR1_-GFP-URA3*  *mrr1*Δ/*MRR1*^F2-1^*-caSAT1* | this study |
| CAG48MRR1M4K3B1 and ‑2 | CAG48MRR1M4B | *MDR1*/*mdr1*::P*_MDR1_-GFP-URA3*  *mrr1*Δ/*MRR1*^F5^*-caSAT1* | this study |
| CAG48MRR1M4K4B1 and ‑2 | CAG48MRR1M4B | *MDR1*/*mdr1*::P*_MDR1_-GFP-URA3*  *mrr1*Δ/*MRR1*^G2-2^*-caSAT1* | this study |
| CAG48MRR1M4K5B1 and ‑2 | CAG48MRR1M4B | *MDR1*/*mdr1*::P*_MDR1_-GFP-URA3*  *mrr1*Δ/*MRR1*^G5^*-caSAT1* | this study |
| CAG48MRR1M2K3B1 and ‑2 | CAG48MRR1M2B | *MDR1*/*mdr1*::P*_MDR1_-GFP-URA3*  *MRR1*/*MRR1*^F5^*-caSAT1* | this study |
| CAG48MRR1M2K5B1 and ‑2 | CAG48MRR1M2B | *MDR1*/*mdr1*::P*_MDR1_-GFP-URA3*  *MRR1*/*MRR1*^G5^*-caSAT1* | this study |

^a^ *SAT1-FLIP* denotes the *SAT1* flipper cassette.

**References**

1. Gillum AM, Tsay EY, Kirsch DR (1984) Isolation of the *Candida albicans* gene for orotidine-5'-phosphate decarboxylase by complementation of *S. cerevisiae ura3* and *E. coli pyrF* mutations. Mol Gen Genet 198: 179-182.
2. Fonzi WA, Irwin MY (1993) Isogenic strain construction and gene mapping in *Candida albicans*. Genetics 134: 717-728.
3. Franz R, Kelly SL, Lamb DC, Kelly DE, Ruhnke M, et al. (1998) Multiple molecular mechanisms contribute to a stepwise development of fluconazole resistance in clinical *Candida albicans* strains. Antimicrob Agents Chemother 42: 3065-3072.
4. Saidane S, Weber S, De Deken X, St-Germain G, Raymond M (2006) *PDR16*-mediated azole resistance in *Candida albicans*. Mol Microbiol 60: 1546-1562.
5. Wirsching S, Michel S, Morschhäuser J (2000) Targeted gene disruption in *Candida albicans* wild-type strains: the role of the *MDR1* gene in fluconazole resistance of clinical *Candida albicans* isolates. Mol Microbiol 36: 856-865.
