# Supplementary material for: The Transcription Factor Mrr1p Controls Expression of the MDR1 Efflux Pump and Mediates Multidrug Resistance in Candida albicans
Source: PLoS Pathog. 2007 Nov 2;3(11):e164. doi: 10.1371/journal.ppat.0030164 (PMC2048531; doi:10.1371/journal.ppat.0030164)
Supplement: Supplementary file 3 [file ppat.0030164.st003.doc]

**Table S3: Allelic differences in the *MRR1* coding region**

| **Allele** |  | **6** | **159** | **457** | **462** | **492** | **512** | **528** | **594** | **888** | **897** | **972** | **999** | **1022** | **1134** | **1437** | **1692** | **1719** | **1776** | **1782** | **1791** |
| --- | --- | --- | --- | --- | --- | --- | --- | --- | --- | --- | --- | --- | --- | --- | --- | --- | --- | --- | --- | --- | --- |
|  |  |  |  |  |  |  |  |  |  |  |  |  |  |  |  |  |  |  |  |  |  |
| *MRR1*^F2-1^ | Nucleotide | A | C | C | A | C | T | T | G | A | T | C | T | T | T | G | G | T | C | T | C |
|  | Amino acid | Ser | Ser | Leu | Gln | Ser | Ser | Thr | Lys | Gly | Ser | Ala | Leu | Val | Thr | Lys | Leu | Tyr | Leu | Ser | Val |
|  |  |  |  |  |  |  |  |  |  |  |  |  |  |  |  |  |  |  |  |  |  |
| *MRR1*^F2-2^ | Nucleotide | G | T | T | G | T | C | C | A | C | C | T | C | A | A | A | A | C | T | T | T |
|  | Amino acid | Ser | Ser | Leu | Gln | Ser | Pro | Thr | Lys | Gly | Ser | Ala | Leu | Glu | Thr | Lys | Leu | Tyr | Leu | Ser | Val |
|  |  |  |  |  |  |  |  |  |  |  |  |  |  |  |  |  |  |  |  |  |  |
| *MRR1*^F5^ | Nucleotide | A | C | C | A | C | T | T | G | A | T | C | T | T | T | G | G | T | C | T | C |
|  | Amino acid | Ser | Ser | Leu | Gln | Ser | Ser | Thr | Lys | Gly | Ser | Ala | Leu | Val | Thr | Lys | Leu | Tyr | Leu | Ser | Val |
|  |  |  |  |  |  |  |  |  |  |  |  |  |  |  |  |  |  |  |  |  |  |
| *MRR1*^G2-1^ | Nucleotide | A | C | C | A | C | T | T | G | A | T | C | T | T | T | G | G | T | C | T | C |
|  | Amino acid | Ser | Ser | Leu | Gln | Ser | Ser | Thr | Lys | Gly | Ser | Ala | Leu | Val | Thr | Lys | Leu | Tyr | Leu | Ser | Val |
|  |  |  |  |  |  |  |  |  |  |  |  |  |  |  |  |  |  |  |  |  |  |
| *MRR1*^G2-2^ | Nucleotide | A | C | T | G | T | C | C | A | C | C | T | C | A | A | A | A | T | T | C | C |
|  | Amino acid | Ser | Ser | Leu | Gln | Ser | Pro | Thr | Lys | Gly | Ser | Ala | Leu | Glu | Thr | Lys | Leu | Tyr | Leu | Ser | Val |
|  |  |  |  |  |  |  |  |  |  |  |  |  |  |  |  |  |  |  |  |  |  |
| *MRR1*^G5^ | Nucleotide | A | C | T | G | T | C | C | A | C | C | T | C | A | A | A | A | T | T | C | C |
|  | Amino acid | Ser | Ser | Leu | Gln | Ser | Pro | Thr | Lys | Gly | Ser | Ala | Leu | Glu | Thr | Lys | Leu | Tyr | Leu | Ser | Val |

| **Allele** |  | **1806** | **1824** | **1851** | **1872** | **2047** | **2157** | **2244** | **2358** | **2370** | **2373** | **2385** | **2394** | **2502** | **2529** | **2538** | **2577** | **2589** | **2595** | **2990** | **3058** |
| --- | --- | --- | --- | --- | --- | --- | --- | --- | --- | --- | --- | --- | --- | --- | --- | --- | --- | --- | --- | --- | --- |
|  |  |  |  |  |  |  |  |  |  |  |  |  |  |  |  |  |  |  |  |  |  |
| *MRR1*^F2-1^ | Nucleotide | T | A | T | A | C | G | A | C | C | C | A | C | G | T | C | G | A | C | G | G |
|  | Amino acid | Ser | Glu | Asn | Leu | Pro | Glu | Pro | Leu | Ala | Gly | Pro | Phe | Val | Asn | His | Ser | Glu | Cys | Gly | Glu |
|  |  |  |  |  |  |  |  |  |  |  |  |  |  |  |  |  |  |  |  |  |  |
| *MRR1*^F2-2^ | Nucleotide | A | G | C | G | C | A | C | C | C | C | A | C | G | T | C | G | A | C | G | C |
|  | Amino acid | Ser | Glu | Asn | Leu | Pro | Glu | Pro | Leu | Ala | Gly | Pro | Phe | Val | Asn | His | Ser | Glu | Cys | Gly | Gln |
|  |  |  |  |  |  |  |  |  |  |  |  |  |  |  |  |  |  |  |  |  |  |
| *MRR1*^F5^ | Nucleotide | T | A | T | A | T | G | A | C | C | C | A | C | G | T | C | G | A | C | G | G |
|  | Amino acid | Ser | Glu | Asn | Leu | Ser | Glu | Pro | Leu | Ala | Gly | Pro | Phe | Val | Asn | His | Ser | Glu | Cys | Gly | Glu |
|  |  |  |  |  |  |  |  |  |  |  |  |  |  |  |  |  |  |  |  |  |  |
| *MRR1*^G2-1^ | Nucleotide | T | A | T | A | C | G | A | C | C | C | A | C | G | T | C | G | A | C | G | C |
|  | Amino acid | Ser | Glu | Asn | Leu | Pro | Glu | Pro | Leu | Ala | Gly | Pro | Phe | Val | Asn | His | Ser | Glu | Cys | Gly | Gln |
|  |  |  |  |  |  |  |  |  |  |  |  |  |  |  |  |  |  |  |  |  |  |
| *MRR1*^G2-2^ | Nucleotide | T | A | T | A | C | A | A | T | T | T | T | T | C | C | T | A | G | T | G | C |
|  | Amino acid | Ser | Glu | Asn | Leu | Pro | Glu | Pro | Leu | Ala | Gly | Pro | Phe | Val | Asn | His | Ser | Glu | Cys | Gly | Gln |
|  |  |  |  |  |  |  |  |  |  |  |  |  |  |  |  |  |  |  |  |  |  |
| *MRR1*^G5^ | Nucleotide | T | A | T | A | C | A | A | T | T | T | T | T | C | C | T | A | G | T | T | C |
|  | Amino acid | Ser | Glu | Asn | Leu | Pro | Glu | Pro | Leu | Ala | Gly | Pro | Phe | Val | Asn | His | Ser | Glu | Cys | Val | Gln |

Only nucleotide positions in which one of the alleles present in isolates F2, F5, G2, and G5 differs from *MRR1* of strain SC5314 (orf19.7372) are shown, the positions with respect to the *MRR1* start codon are given on top. Black: no substitution; green: conservative substitution, blue: non-conservative substitution, red: gain-of-function mutations in the *MRR1* alleles of the resistant isolates F5 and G5.
