# Supplementary material for: The Transcription Factor Mrr1p Controls Expression of the MDR1 Efflux Pump and Mediates Multidrug Resistance in Candida albicans
Source: PLoS Pathog. 2007 Nov 2;3(11):e164. doi: 10.1371/journal.ppat.0030164 (PMC2048531; doi:10.1371/journal.ppat.0030164)
Supplement: Supplementary file 4 [file ppat.0030164.st005.doc]

**Table S5: Primers used in this study**

| Primer | Sequence ^a^ |
| --- | --- |
| ZCF36-1 | 5`-TCTAAGTCGACAAAAATGTCAATTGCCACCACCC-3` |
| ZCF36-2 | 5`-GCCAATTCACCAGATCTAATTTAATTGG-3` |
| ZCF36-3 | 5`-GAATAATTCGGAGCTCAATTTGCGTTTAGCC-3` |
| ZCF36-4 | 5`-GGTGTTTCTATAGCCGCGGTGGCAATTGAC-3` |
| ZCF36-5 | 5`-TAACACTCGAGAAGCTGATCTTAGTTCATTTG-3` |
| ZCF36-6 | 5`-ATATTGGGCCCGCTACCATAAGCCTCGCTCG-3` |
| ZCF36-7 | 5`-CCAAATGCATTGATTCTGGTGAATTGGCAGC-3` |
| ZCF36-8 | 5`-AATCGGATCCATTGGTAAAAAGTTGATCAAATGG-3` |
| ZCF36seq1 ^b^ | 5`-ATTACAATGTGTCCCACACACG-3` |
| ZCF36seq2 ^b^ | 5`-CAGTTTACTTTATCCATTTATGCC-3` |
| ZCF36seq3 ^b^ | 5`-AGTTCCATTTATAGAAGAAGGC-3` |
| ZCF36seq4 ^b^ | 5`-GTTGGAATTGCAGCTGTATCC-3` |
| ZCF36seq5 ^b^ | 5`-CCCTTGGTAATCTTCCACGC-3` |
| ZCF36seq6 ^b^ | 5`-CATCCTTGTATTCCGTTTCACC-3` |
| ZCF36seq7 ^b^ | 5`-GAAGTCACTATTACGTTGAGTG-3` |
| ZCF36seq8 ^b^ | 5`-CTTGATTGATAAGAGTTTGGATC-3` |
| MDR1F | 5`-ACATAAATACTTTGCCCATCCAGAA-3` |
| MDR1R | 5`-AAGAGTTGGTTTGTAATCGGCTAAA-3` |
| 18SF | 5`-CACGACGGAGTTTCACAAGA-3` |
| 18SR | 5`-CGATGGAAGTTTGAGGCAAT-3` |

^a^ Restriction sites introduced into the primers are underlined.

^b^ Primers used for sequencing of PCR-amplified *MRR1* fragments
